# Concentrated oat β-glucan, a fermentable fiber, lowers serum cholesterol in hypercholesterolemic adults in a randomized controlled trial

**DOI:** 10.1186/1475-2891-6-6

**Published:** 2007-03-26

**Authors:** Katie M Queenan, Maria L Stewart, Kristen N Smith, William Thomas, R Gary Fulcher, Joanne L Slavin

**Affiliations:** 1 Department of Food Science and Nutrition, University of Minnesota, St. Paul, MN, USA; 2 Division of Biostatistics, School of Public Health, University of Minnesota, Minneapolis, MN, USA; 3 Department of Food Science, University of Manitoba, Winnipeg, Manitoba, Canada

## Abstract

**Background:**

Soluble fibers lower serum lipids, but are difficult to incorporate into products acceptable to consumers. We investigated the physiological effects of a concentrated oat β-glucan on cardiovascular disease (CVD) endpoints in human subjects. We also compared the fermentability of concentrated oat β-glucan with inulin and guar gum in a model intestinal fermentation system.

**Methods:**

Seventy-five hypercholesterolemic men and women were randomly assigned to one of two treatments: 6 grams/day concentrated oat β-glucan or 6 grams/day dextrose (control). Fasting blood samples were collected at baseline, week 3, and week 6 and analyzed for total cholesterol, HDL cholesterol, LDL cholesterol, triglycerides, glucose, insulin, homocysteine and C-reactive protein (CRP). To estimate colonic fermentability, 0.5 g concentrated oat β-glucan was incubated in a batch model intestinal fermentation system, using human fecal inoculum to provide representative microflora. Fecal donors were not involved with the β-glucan feeding trial. Inulin and guar gum were also incubated in separate serum bottles for comparison.

**Results:**

Oat β-glucan produced significant reduction from baseline in total cholesterol (-0.3 ± 0.1 mmol/L) and LDL cholesterol (-0.3 ± 0.1 mmol/L), and the reduction in LDL cholesterol were significantly greater than in the control group (p = 0.03). Concentrated oat β-glucan was a fermentable fiber and produced total SCFA and acetate concentrations similar to inulin and guar gum. Concentrated oat β-glucan produced the highest concentrations of butyrate at 4, 8, and 12 hours.

**Conclusion:**

Six grams concentrated oat β-glucan per day for six weeks significantly reduced total and LDL cholesterol in subjects with elevated cholesterol, and the LDL cholesterol reduction was greater than the change in the control group. Based on a model intestinal fermentation, this oat β-glucan was fermentable, producing higher amounts of butyrate than other fibers. Thus, a practical dose of β-glucan can significantly lower serum lipids in a high-risk population and may improve colon health.

## Background

Cardiovascular disease (CVD) remains the top cause of death in the United States, with saturated fat and trans fat intake, serum cholesterol, and obesity as major risk factors. Diets recommended for improvement of cardiovascular risk factors include a diet high in dietary fiber. Cereal fibers that are high in water-soluble fiber, such as β-glucan, may improve cardiovascular disease risk through improvements in serum cholesterol and other intermediary risk factors. Oats are high in soluble fiber and appear to reduce CVD risk when consumed as part of a moderate fat, balanced diet. Soluble fiber β-glucan is thought to be the active component for the cholesterol lowering effect of oats. Few studies have been conducted on the effectiveness of concentrated β-glucan from oats and changes in physiological endpoints.

Concentrated β-glucan from oat is now commercially available as a dietary supplement. Oat β-glucan is a natural polymer comprised of individual glucose molecules that are linked together by a series of β-(1–3) and β-(1–4) linkages. Beta-glucan was identified as the major fermentable component in both cooked and uncooked rolled oats; the beta-glucan was not affected by cooking [1]. Rolled oats and isolated beta-glucan (termed oat gum in the report) contributed to increased gut viscosity in male Wistar rats [2].

The hypothesis that soluble dietary fiber lowers blood cholesterol levels by interfering with the absorption of dietary cholesterol is appealing. Increased gut viscosity may prevent dietary cholesterol from reaching the intestinal epithelium. However, it is unlikely that fiber-induced hypocholesterolemia is solely a result of this mechanism [3]. Exogenenous cholesterol represents only about one quarter of the body's cholesterol, and a significant change in absorption would lead to even smaller changes in blood cholesterol levels. Some fibers may decrease absorption of dietary cholesterol by altering the composition of the bile acid pool. Pectin and oat bran increased the portion of the total bile acid pool that was deoxycholic acid (DCA) [4]. DCA has been noted to decrease the absorption of exogenous cholesterol in humans [5]. Fermentation products may also alter cholesterol metabolism. Propionate is thought to suppress cholesterol synthesis, but some results have been inconclusive [6-10]. Acetate produced by the fermentation of oat bran may also contribute to cholesterol lowering [11].

Studying gut effects of fiber in humans is difficult due to the invasive and expensive nature of colonic observation as well as the dynamic nature of the colon. Excreted colon contents will not necessary represent colon contents in the proximal or even distal colon, due to continual fermentation of fiber and continual absorption of minerals and SCFA across the epithelium. In vitro fermentation is a noninvasive, time-efficient means to estimate fiber fermentability.

The cholesterol lowering effect of whole oats has been well documented and the United States FDA issued a health claim on oats for the prevention of cardiovascular disease in 1996 [12]. However, limited research has been conducted with oat β-glucan separated from the oat grain. This current paper aims to determine if the beneficial physiological effects of oats can be seen with concentrated oat β-glucan.

A barrier to use of soluble fiber in the past has been the poor acceptability of foods high in soluble fiber for an extended period. We measured the effect of 6 g/day of concentrated β-glucan from oat on biomarkers of interest in CHD and CVD. The human feeding trial was designed to identify if the physiological effects of concentrated β glucan from oat will improve serum biomarkers of cardiovascular risk in subjects at high risk of cardiovascular disease by measuring changes in total cholesterol, LDL-cholesterol, HDL-cholesterol, triglycerides, apolipoprotein A-1, apolipoprotein B and other blood metabolites. Model intestinal fermentation was used to estimate in vivo short-chain fatty acid production.

## Methods

Ninety hypercholesterolemic subjects were recruited for the study via flyers posted on the University of Minnesota campus and newspaper advertisements in the University of Minnesota Daily newspaper. To be included in the study, subjects needed to be healthy, non-smoking men and women between the ages of 22 and 65 years at risk for CVD (as defined by a total cholesterol greater than 200 mg/dl), with spoken and written English literacy. Subjects exclusion criteria included BMI > 30 upon admission to study; CVD, Diabetes Mellitus (fasting blood sugar > 126 mg/dl), chronic inflammatory diseases (e.g. Crohn's, rheumatoid arthritis), cancer in prior 5 years, renal or hepatic disease, recent bacterial infection (< 2 weeks), acute febrile illness in prior 2 months, history of drug or alcohol abuse in prior 6 months, lipid-lowering, anti-hypertensive or anti-inflammatory steroid medication use, active weight loss > 5 kg in prior 3 months (intended or unintended), concurrent or recent (within 30 days) intervention study participation. The inclusion and exclusion criteria were assessed via telephone screening, a health history questionnaire, and the initial total cholesterol screening visit. Subjects received oral and written information about the study, including a comprehensive study newsletter and individual written consent was obtained from each subject.

Subjects were asked to consume their usual diet with the addition of either a placebo supplement or dietary fiber from oat. The risks in the study were minimal. Subjects maintained their usual activities during the study with periodic visits for information sessions, sample collections, and supplement pickup.

### Study design

The University of Minnesota Institutional Review Board Human Subjects Committee approved all aspects of this research. The study was a randomized, double-blind parallel group design. A total of 90 patients were enrolled with 45 patients per treatment arm. Subjects were randomly assigned to either placebo or treatment, stratified by age and sex. Fifteen subjects (n = 10 treatment, n = 5 placebo) were excluded from final analysis because their baseline cholesterol value was below 200 mg/dl despite a screening value above 200 mg/dl. Treatment was 6 grams of concentrated β-glucan from oat per day (from 12 g oat bran concentrate containing 54% oat β glucan). Placebo was 6 grams of dextrose monohydrate per day. Both were delivered as dietary supplements in the form of a powder. Subjects mixed their supplement with a beverage using a study-provided electric hand blender (Braun). Subjects were instructed to take the supplement via oral administration with their morning and evening meal for six weeks.

### Study visits

Subjects attended an initial pre-screening visit to verify eligibility for the study (visit 1). Subjects who met study criteria were scheduled for a baseline visit, one day before the intervention began (visit 2). Visits 3 and 4 were scheduled for day 21 (midpoint) and day 42 (final) of the intervention.

### Diet records

Three-day diet records were obtained from each subject at the midpoint and final visits. Subjects recorded two weekdays and one weekend day of their total food intake. All records were analyzed for total nutrient composition of each day's intake using the First DataBank Nutritionist Five software. Total calories, carbohydrate, fat, protein, cholesterol, saturated fat, monounsaturated fat, polyunsaturated fat, total fiber, soluble fiber, insoluble fiber and crude fiber were analyzed for each subject for average intake. Analysis of fiber did not include the addition of oat β-glucan to the diet.

### Blood samples

At each visit subjects were asked to fast overnight for a minimum of 10 hours and then have blood samples drawn at the General Clinical Research Center, University of Minnesota, Twin Cities. Blood was drawn for the following measurements: plasma total cholesterol, triglycerides, HDL cholesterol, LDL cholesterol, apolipoprotein A-I, apolipoprotein B, complete blood count and differential, homocysteine, c-reactive protein and comprehensive metabolic panel. Plasma LDL cholesterol concentration was calculated using the Friedewald equation. All specimens collected were transported via courier to Quest Diagnostics (Wood Dale, IL) for analysis.

### Anthropometric measurements

Blood pressure was measured at each visit for each subject using a standardized sphygmomanometer (Tycos). Three measurements were taken, one minute apart, with the subject in a semi-reclined position following the approved National Institutes of Health method [13]. These three values were averaged individually for diastolic and systolic pressures. Standing weight was recorded every visit to the nearest 1/4 lb with light clothing and no shoes on a calibrated balance beam scale.

### Symptom survey

Each visit participants completed a short survey rating gastrointestinal effects on a scale related to normal. Symptoms recorded include: frequency of stools, consistency of stools, degree of intestinal bloating and degree of flatulence.

### Model intestinal fermentation

Concentrated oat β-glucan (54% β-glucan), partially hydrolyzed guar gum (MW 400 kDa), and inulin (90% dp = 10, 10% dp = 1–2) were subjected to model intestinal fermentation [14]. Glucose served as a positive control because it is fully fermentable by colonic bacteria. No fiber was the negative control to assess the concentrations of SCFA produced from substrates residing in the fecal inoculum. Chemical reagents were obtained from Fisher Scientific (New Hampton, NH, USA), Sigma Aldrich (St. Louis, MO, USA), and VWR Scientific (West Chester, PA, USA).

Briefly, the fibers were hydrated for 12 hours in 40 ml sterile trypticase peptone media fortified with minerals, phosphate buffer, and a reducing solution (950 ml distilled water, 6.25 g cysteine hydrochloride, 40 ml 1N NaOH, 6.25 g sodium sulfide monohydrate) at 4°C. Five-100 mL serum bottles were prepared for each fiber sample (0.5 g), one for each of the five time points: 0, 4, 8, 12, and 24 hours. Control bottles containing either 0.5 g glucose or no added carbohydrate were prepared in the same manner. Two hours prior to inoculation with fecal solution, sample bottles were warmed to 37°C. Fecal inoculum was prepared as described by McBurney and Thompson [14]. Fecal samples from three human subjects consuming a nonspecified Western diet were pooled (125 g total) and diluted with 400 ml distilled water. The solution was homogenized in a blender. Reducing solution was added to the fecal inoculum to obtain a ratio of 15 parts fecal inoculum to 2 parts reducing solution [15].

Ten milliliters of fecal inoculum was added into each serum bottle along with 0.8 mL Oxyrase^® ^oxygen reducing enzyme (Oxyrase Inc., Mansfield, OH). The bottles were immediately flushed with carbon dioxide gas to eliminate oxygen and generate anaerobic conditions. The bottles were gently shaken in a 37°C water bath. One sample bottle for each fiber was removed at 0, 4, 8, 12, and 24 hours. Immediately upon removal, 1 mL of copper sulfate (200 g/L) was added to each bottle to kill the bacteria and cease fermentation. Two-2 ml aliquots were removed. Samples were prepared for gas chromatography as described previously [16,17].

Lactate, acetate, propionate, butyrate isobutyrate, 2-methylbutyrate, isovalerate, and valerate were determined by gas chromatography using a Hewlett Packard 6890 gas chromatograph (Hewlett Packard, Palo Alto, CA) with a 4% carbowax 20 M/80/120 carbopack B-DA column (Supleco, Bellefonte, PA) a temperature of 175 degrees C. Flow rates for nitrogen, hydrogen, and air were 24, 40, and 450 mL/min, respectively. All SCFA concentrations were corrected for the control concentration of SCFA at each time point. Molar ratios of SCFA were determined by dividing the number of moles of each SCFA (acetate, propionate, and butyrate) by the total moles of acetate, propionate, and butyrate.

### Statistical analysis

Data was reported as means ± SEM. Changes were calculated by subtracting baseline from the 6-week value, so that positive changes indicate an increase. Baseline values and changes were compared between oat β-glucan and control by two-sample t-tests, while paired t-tests were used for within-group changes from baseline. In the model intestinal fermentation, all SCFA concentrations were corrected for the negative control concentration of SCFA at each time point. SCFA concentrations were compared between fibers at each time point separately, using Tukey pairwise procedure for multiple comparisons. Statistical analyses were performed with SAS statistical software package, versions 8.0 and 9.1 (SAS Institute Inc, Cary, NC, USA).

## Results

### Human trial

Demographic and clinical characteristics of the oat β-glucan and control groups did not differ at baseline (Table 1). There were no changes from baseline in dietary intake, weight, or blood pressure in either group (Table 2). Total cholesterol significantly decreased within the treatment group (p < 0.025). Total cholesterol decreased 0.3 ± 0.1 mmol/L (mean ± SEM) in subjects consuming oat β-glucan. Total cholesterol did not significantly change within the placebo group. Total cholesterol decreased 0.1 ± 0.08 mmol/L (mean ± SEM) within the placebo group, but this change was not significant. Total cholesterol did not significantly change between groups.

**Table 1:** Baseline Demographic and Clinical Data

	**Treatment**	**Placebo**	**P value**
n (female/male)	35 (22/13)	40 (28/12)	0.513
Age (yr)	44.5 ± 2.2	45.3 ± 2.0	0.796
Blood pressure (systolic/diastolic, mmHg)	121/69 ± 2.2/1.4	121.6/67.1 ± 1.9/1.5	0.868/0.760
Weight (kg)	81.8 ± 2.8	79.6 ± 2.9	0.598
Total Cholesterol (mmol/L)	6.2 ± 0.1	6.2 ± 0.1	0.976
Triglyceride (mmol/L)*	1.9 ± 0.1	1.9 ± 0.2	0.989
HDL cholesterol (mmol/L)	1.4 ± 0.06	1.3 ± 0.05	0.806
LDL cholesterol (mmol/L)**	4.1 ± 0.1	4.2 ± 0.1	0.651
Cholesterol/HDL ratio	4.8 ± 0.2	4.8 ± 0.2	0.981
C-Reactive protein (mg/dL)	0.35 ± 0.08	0.38 ± .06	0.817
Homocysteine (μmol/L)	8.0 ± 0.3	7.9 ± 0.3	0.861
Insulin (pmol/L)	56.2 ± 4.2	68.1 ± 9.0	0.273
Glucose (mmol/L)	5.4 ± 0.3	5.3 ± 0.1	0.840

Data are means ± SEM.

* Excludes one subject in the Placebo group with fasting triglycerides > 400 mg/dL.

** Excludes two subjects in the Placebo group with fasting triglycerides > 400 mg/dL

**Table 2:** Comparison of endpoints at six weeks expressed as changes from baseline.

	**Treatment**	**Placebo**	**P value**
Total Cholesterol (mmol/L)	-0.3 ± 0.1 ***	-0.1 ± 0.08	0.184
LDL cholesterol (mmol/L)**	-0.3 ± 0.1 ***	-0.04 ± 0.08	0.026
HDL cholesterol (mmol/L)	-0.02 ± 0.02	-0.009 ± 0.02	0.842
Triglyceride (mmol/L)*	0.09 ± 0.1	-0.2 ± 0.1***	0.030
Cholesterol/HDL ratio	-0.2 ± 0.1	-0.1 ± 0.1	0.947
C-Reactive protein (mg/dL)	-0.03 ± 0.06	0.01 ± 0.04	0.546
Homocysteine (μM/L)	0.4 ± 0.4	0.2 ± 0.2	0.528
Insulin (pmol/L)	6.9 ± 4.9	4.2 ± 10.4	0.804
Glucose (mmol/L)	0.2 ± 0.09	0.03 ± 0.09	0.259
Weight (kg)	-0.7 ± 0.3	1.4 ± 1.3	0.117
Blood pressure (systolic/diastolic, mmHg, value at end point)	119/69 ± 1.9/1.3	119/69 ± 2.0/1.7	0.832/0.856
Total Energy Intake per day (kJ)	-519 ± 330	-343 ± 360	0.724
Carbohydrate intake (% of total)	0.4 ± 2.0	1.8 ± 1.3	0.535
Protein Intake (% of total)	0.9 ± 0.6	-0.9 ± 1.1	0.041
Fat Intake (% of total)	-0.7 ± 1.8	-1.2 ± 1.1	0.784
Dietary Fiber Intake (grams)	-0.3 ± 1.1	-1.4 ± 1.6	0.454

Data are means ± SEM.

***Statistically different from baseline, within group comparison p < 0.05

LDL cholesterol significantly decreased within the treatment group (p < 0.025). LDL cholesterol dropped 0.3 ± 0.1 mmol/L (mean ± SEM) in subjects consuming oat β-glucan. LDL cholesterol did not change significantly within the placebo group; the mean decrease was 0.04 mmol/L. Treatment of oat β-glucan significantly lowered LDL cholesterol compared to control (p < 0.026). HDL cholesterol did not change significantly in either the treatment or placebo group.

Triglycerides increased 0.09 ± 0.1 mmol/L (mean ± SEM) in the treatment group, but this change was not significantly different than 0. Triglycerides fell 0.2 ± 0.1 mmol/L (mean ± SEM) in the placebo group. The triglyceride change between the treatment and control group was significantly different (p = 0.030).

The oat β-glucan group experienced significant reduction from baseline in total cholesterol and LDL cholesterol, and the reduction in LDL cholesterol was greater than in the control group. Triglycerides were reduced in the control group but not the oat β-glucan group. Neither group showed changes in HDL cholesterol, C-reactive protein, homocysteine, insulin, or glucose.

Both groups reported no change in stool consistency, stool frequency, and bloating during the study, although the oat β-glucan group reported increased flatulence. Three-day food records completed on days 1–3, 22–24, and 39–41 were analyzed to verify dietary intake did not significantly change during the study. Changes in energy intake and diet composition are shown in Table 2.

### Model intestinal fermentation

Concentrations of total short-chain fatty acids (TSCFA), acetate, propionate, and butyrate produced during the model intestinal fermentation are shown in Table 3. Concentrated oat β-glucan and inulin produced similar concentrations of TSCFA and were both significantly greater than guar gum at 4 hours. Oat β-glucan TSCFA production increased from 0 to 8 hours, and remained constant from 8 to 24 hours. Total short-chain fatty acid production by inulin peaked at 8 hours and decreased until 24 hours. Guar gum produced increasing concentrations of TSCFA at all time points. At 24 hours, guar gum produced the greatest concentrations of TSCFA while oat-β glucan and inulin produced significantly few TSCFA.

**Table 3:** Total SCFA^†^, acetate, propionate, and butyrate production^‡ ^(μmol/ml) of isolated oat β-glucan (54% soluble fiber), guar gum sample (MW 400 kDa) and inulin sample (degree of polymerization 100% ≈10) after 0, 4,8,12, and 24 hours in a model intestinal fermentation, analyzed by gas chromatography.

Sample		Oat β-glucan	Guar Gum	Inulin
Total SCFA	0 h	0.6 ± 0.2	-0.7 ± 0.5	-0.3 ± 0.2
	4 h	32.7 ± 0.3^a^*	7.7 ± 0.02^b^	33.8 ± 0.8^a^
	8 h	50.5 ± 4.0^a^	45.7 ± 3.2^a^	75.4 ± 0.7^b^
	12 h	50.3 ± 6.2	62.0 ± 0.6	55.6 ± 1.8
	24 h	48.7 ± 1.5^a^	68.6 ± 0.8^b^	51.8 ± 0.8^a^

Acetate	0 h	-0.4 ± 0.01	-0.2 ± 0.05	-0.2 ± 0.02
	4 h	23.1 ± 0.9^a^	6.6 ± 0.5^b^	23.3 ± 0.4^a^
	8 h	40.0 ± 3.1^a^	34.2 ± 2.2^a^	63.7 ± 1.8^b^
	12 h	37.5 ± 4.5	45.5 ± 0.1	48.2 ± 4.2
	24 h	24.7 ± 1.7^a^	34.6 ± 0.2^b^	30.3 ± 0.8^ab^

Propionate	0 h	-0.1 ± 0.01	0.05 ± 0.04	-0.04 ± 0.01
	4 h	0.7 ± 0.04	0.5 ± 0.1	0.4 ± 0.1
	8 h	0.8 ± 0.7^a^	4.5 ± 0.8^b^	-2.2 ± 0.1^a^
	12 h	3.8 ± 1.2^a^	16.8 ± 0.3^b^	-6.2 ± 0.2^c^
	24 h	11.9 ± 0.004^a^	28.5 ± 0.7^b^	0.08 ± 0.1^c^

Butyrate	0 h	-0.1 ± 0.02	-0.03 ± 0.04	-0.03 ± 0.02
	4 h	0.4 ± 0.02	0.2 ± 0.05	0.2 ± 0.04
	8 h	2.2 ± 0.3^a^	0.9 ± 0.2^b^	0.6 ± 0.05^b^
	12 h	7.7 ± 0.9^a^	3.3 ± 0.1^b^	-.004 ± 0.1^c^
	24 h	13.3 ± 0.3^a^	6.8 ± 0.1^b^	23.1 ± 0.1^c^

^† ^Total SCFA = acetate, propionate, butyrate, isobutyrate, 2-methylbutyrate, isovalerate, lactate, and valerate (if detectable).

^‡^Values shown are mean ± standard error, each from 2 samples

*Mean production of each compound was compared at each time point between oat β-glucan, guar gum, and inulin. Within each row, means that do not share a superscript letter are significantly different (p < 0.05), and means that share a letter are not significantly different.

Acetate was the major contributor to TSCFA concentrations at all time points by all fibers. Acetate production mimicked the TSCFA production patterns with oat β-glucan and inulin being the fermented more rapidly than guar gum between 0 and 4 hours. At 8 hours, inulin produced significantly more acetate than oat β-glucan and guar gum. Acetate production at 12 hours was not different between the three fibers. At 24 hours, oat β-glucan produced the lowest concentrations of acetate, although this difference was only significant between guar gum and oat-β glucan.

Propionate was the SCFA produced in the second greatest concentration by oat β-glucan and guar gum. Oat β-glucan produced little propionate between 0 and 8 hours. From 8 to 24 hours, oat β-glucan produced propionate at a constant rate. Concentrations of propionate produced by oat β-glucan were less than guar gum but greater than inulin from 8 to 24 hours.

Oat-β glucan produced the greatest butyrate concentrations from 4 to 12 hours. Between 12 and 24 hours, butyrate production by inulin increased dramatically, resulting in the greatest butyrate production at 24 hours. Oat β-glucan produced the second greatest concentration of butyrate at 24 hours, with guar gum producing the least butyrate.

## Discussion

Large, prospective, epidemiologic studies find a protective effect of dietary fiber against coronary heart disease and form the basis for recommendations from the National Academy of Science for fiber intake (38 and 25 grams per day for young men and women)[18]. It has been proposed that a protective effect of soluble dietary fiber against CVD is mediated through direct or indirect effects on serum lipids. This study supports that oat β-glucan's role in CVD protection is through its effects on serum lipids. Virtually all other markers tested, other than serum lipids and apo B, did not significantly change as a result of 6 grams concentrated β-glucan.

*Dietary fiber*, as defined by the Institute of Medicine, consists of nondigestible carbohydrates and lignin that are intrinsic and intact in plants [19]. *Functional fiber *consists of concentrated, nondigestible carbohydrates that have beneficial physiological effects in humans [19]. The IOM recommended the elimination of the terms *soluble *and *insoluble*. New terms, *viscous *and *fermentable*, were recommended to describe the physicochemical properties of fibers. *Viscous *fibers such as β-glucan and guar gum have been shown to decrease blood LDL-cholesterol concentrations in animal models [20] and clinical intervention studies [21]. We have shown that consumption of oat β-glucan as a functional fiber has similar physiological effects as oat β-glucan intact in oats.

The reduction of cholesterol is possibly a sum of several effects. It is commonly accepted however that the majority of effect is due to decreased absorption of bile acids. This causes a removal of steroids from the body by fecal excretion resulting in increased catabolism of cholesterol, an increase in the secretion of bile acids, a decrease in lipoprotein cholesterol secretion, and a reduction in the total body pool of cholesterol [22]. Viscous fibers such as oat β-glucan can interfere with the absorption of dietary fat and cholesterol as well as enterohepatic recirculation of cholesterol and bile acids.

Viscous fibers may also delay the gastric emptying of ingested foods into the small intestine, resulting in a sensation of fullness, which may help weight control. Delayed gastric emptying may also reduce postprandial blood glucose concentrations leading to greater insulin sensitivity. In this study however, subjects consuming oat β-glucan or placebo had no change in weight. Perhaps a six week study is too short to impact subjects' weight. In addition, the oat β-glucan had no effect on fasting glucose or insulin concentrations.

Dietary fiber intake may also displace saturated fat intake and thus reduce CVD events [23]. However, three-day food records show no significant difference in saturated fat between treatment and placebo groups – supporting the idea that dietary fiber has beneficial cardiovascular effects independent of saturated fat.

The decrease in LDL cholesterol (0.3 mmol/L) as a result of oat β-glucan administration was a modest decrease. Statin treatment has been shown to decrease LDL cholesterol by 1.3 mmol/L over the course of a six week intervention [24]. However, the decrease in LDL cholesterol as a result of concentrated oat β-glucan administration is large enough to be clinically relevant. A 0.26 mmol/L increase in LDL cholesterol results in a 12% increase in risk of CVD [25].

Dietary interventions, such as The Portfolio diet which is high in plant sterols, soy protein, almonds, and viscous fiber, have been documented to reduce cholesterol levels as effectively as statin therapy [26]. The 12 gram dose of oat β-glucan used in this study was an acceptable dose to the subjects and provided clinically significant changes in cholesterol levels. The effectiveness of concentrated oat β-glucan as an add-on to statin therapy is beyond the scope of this study, but should be considered for future research. Concentrated oat β-glucan would be suitable as a stand-alone supplement to lower cholesterol. This product could also be used as a food ingredient to increase fiber content of food. When incorporated into minimally processed, low-fat food, concentrated oat β-glucan is hypothesized to retain is hypocholesterolemic effects.

This study also estimated the fermentability of concentrated oat β-glucan in a model intestinal fermentation system. Studying colonic fermentation in healthy humans is very difficult. The colonic epithelium is a dynamic system which is continually absorbing SCFAs as they are being produced by colonic bacteria. Currently, no method exists to measure SCFA absorption across the epithelium in humans. Short-chain fatty acid concentration in feces is a reasonable measurement of colonic fermentability. However, model intestinal fermentation is a more rapid, non-invasive, method of assessing fermentability and results from this method correspond with fermentability *in vivo *[27,28].

Colonic fermentation varies between individuals as a result of species of gut bacteria and intestinal transit time. Absorption varies between individuals due to colonic retention time and other metabolites present in the feces, such as bicarbonate or sodium. The batch fermentation system eliminates differences in SCFA production due to the aforementioned variables and allows several fibers to be compared subjectively. Short-chain fatty acid production may play a role in decreasing cholesterol, but acetate and propionate concentrations were not remarkably different than concentrations produced by inulin or guar gum. Other mechanisms discussed above likely contribute to the cholesterol lowering feature of oat β-glucan.

The data from this study supports numerous studies showing increased intakes of viscous fiber decrease blood LDL-cholesterol concentrations [21]. Insulin concentrations, glucose concentrations, weight, homocysteine and CRP however did not significantly change in response to oat β-glucan and lead us to the question "Is this functional fiber different than dietary fiber?" It is likely that some food constituents, such as vitamins, trace elements, phenolic compounds, and phytoestrogens, found in fiber-rich foods also affect CVD risk and operate via pathways other than the lipid-regulating pathway [29]. The functional fiber, oat β-glucan, may be beneficial to subjects with high CVD risk because of its ability to significantly lower LDL cholesterol concentrations. However, several other mechanisms may underlie the cardiovascular benefits of dietary fiber. These include improvement in postprandial glucose and insulin responses and lowering of blood pressure and body weight [30]. Thus, the present study suggests that oat β-glucan significantly lowers LDL-cholesterol concentrations in subjects with high CVD risk.

## Conclusion

A dosage of 6 grams concentrated β-glucan per day for six weeks produced significant reduction from baseline in total and LDL cholesterol. The reduction in LDL cholesterol was significantly greater than in the control group. These results indicate that concentrated β-glucan may be beneficial to high cardiovascular disease risk populations. Concentrated oat β-glucan is a fermentable fiber, with SCFA production similar to inulin and guar gum, as shown in model intestinal fermentation. This source of fiber will likely increase SCFA concentrations in the human colon, in addition to lowering LDL cholesterol.

## Competing interests

The author(s) declare that they have no competing interests.

## Authors' contributions

KMQ carried out the human study design, data review, and contributed to the manuscript. MLS carried out the model intestinal fermentation study, conducted statistical analysis, and contributed to the manuscript. KNS carried out the human study design and data review. WT served as the statistical advisor. GF participated as a co-principal investigator. JLS carried out the human study design and participated as a co-principal investigator.
